# Supplementary material for: HDAC Screening Identifies the HDAC Class I Inhibitor Romidepsin as a Promising Epigenetic Drug for Biliary Tract Cancer
Source: Cancers (Basel). 2021 Jul 31;13(15):3862. doi: 10.3390/cancers13153862 (PMC8345689; doi:10.3390/cancers13153862)
Supplement: Supplementary file 1 [file cancers-13-03862-s001.zip › cancers-1292273-supplementary.pdf]

cancers

MDPI

Supplementary Files

HDAC Screening Identifies the HDAC Class I Inhibitor Romidepsin as a Promising Epigenetic Drug for Biliary Tract Cancer

Christian Mayr, Tobias Kiesslich, Sara Erber, Dino Bekric, Heidemarie Dobias, Marlena Beyreis, Markus Ritter, Tarkan Jäger, Bettina Neumayer, Paul Winkelmann, Eckhard Klieser, Daniel Neureiter

Gene Name	*Forward* Primer (5‘-3‘)	*Reverse* Primer (5‘-3‘)
HDAC1	GGATACGGAGATCCCTAATG	CGTGTTCTGGTTAGTCATATTG
HDAC2	GGTCATGCTAAATGTGTAGAAG	GTCTGGTCCAAAATACTCAAAG
HDAC3	AAAACCTGAAGATGCTGAAC	CCTGCTATAGTTCTCCTCAG
HDAC4	AAAAGAGACCAGATGAGGAG	AGACAGACAGACAAGAGAAC
HDAC5	AGAATGGATTTGCCATCATC	TGGCTACAGAGTTGAAGAAG
HDAC6	AAAAGGAAGAGCTGATGTTG	GATGCAGATAAACTGAGTCG
HDAC7	CTTTCAGGATAGTCGTGATG	ATTTGGCAGAAACATGGTAG
HDAC8	CAGCATAGGTCCTGATTATG	TCCCTTTGATGTAGTTGAGG
HDAC9	AAAGAGAGAATTTCACCTGG	GAAAGCAGTTTGTTCATGTG
HDAC10	GATATCACATTGGTTCTGCC	ATCCCATCTAAGAGGTACAG
HDAC11	TAATGAGCTCAAGAGGAAGG	TCAAACAGAAACTTGATGGC
beta-actin	GACGACATGGAGAAAATCTG	ATGATCTGGGTCATCTTCTC

Figure S1. Primer sequences used for evaluation of HDAC expression in biliary tract cancer cells (all purchased at Merck; KiCqStart® SYBR® Green Primers).

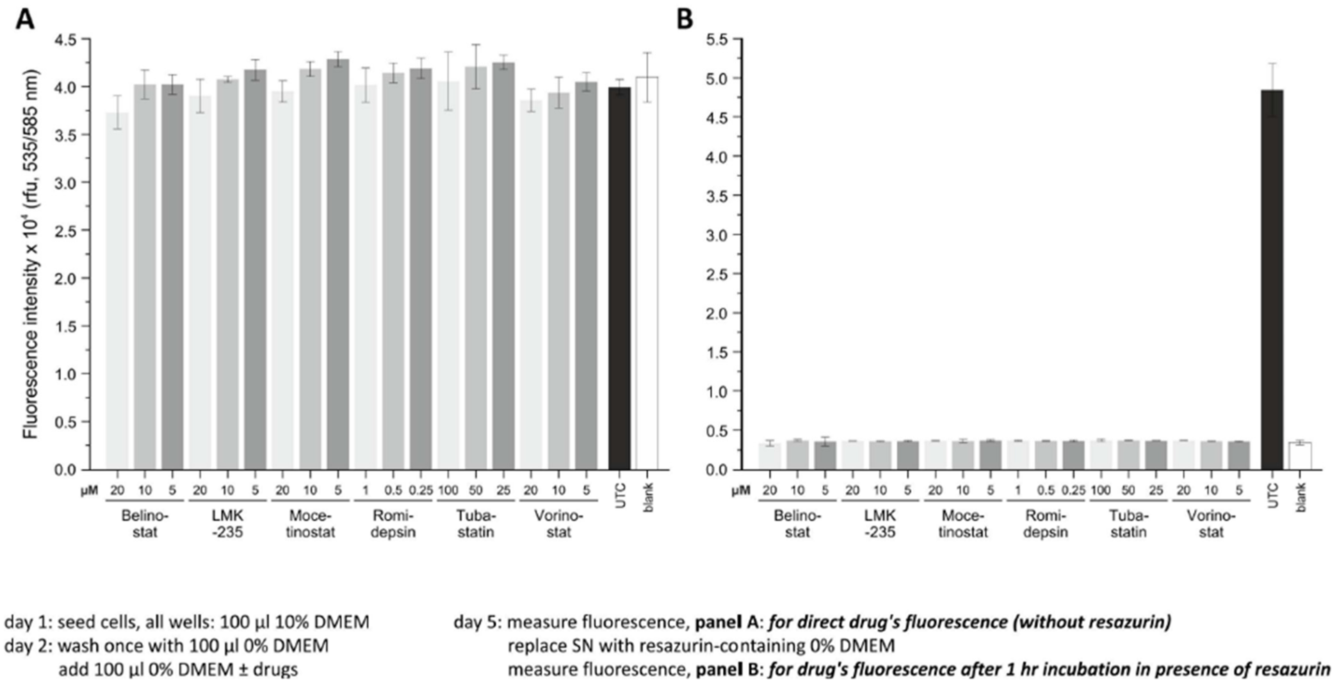

Figure S2. Analysis of autofluorescence of HDAC inhibitors after 72 h. (**A**) Measurement of direct drug's fluorescence (without addition of resazurin) of various concentrations (including the highest used concentration) of the used HDAC inhibitors compared to untreated cells (UTC) and blank. (**B**) Fluorescence of HDAC inhibitors compared to untreated cells and blank using the resazurin assay. Data are presented as mean value of n ≥ 4 technical replicates ± standard deviation.

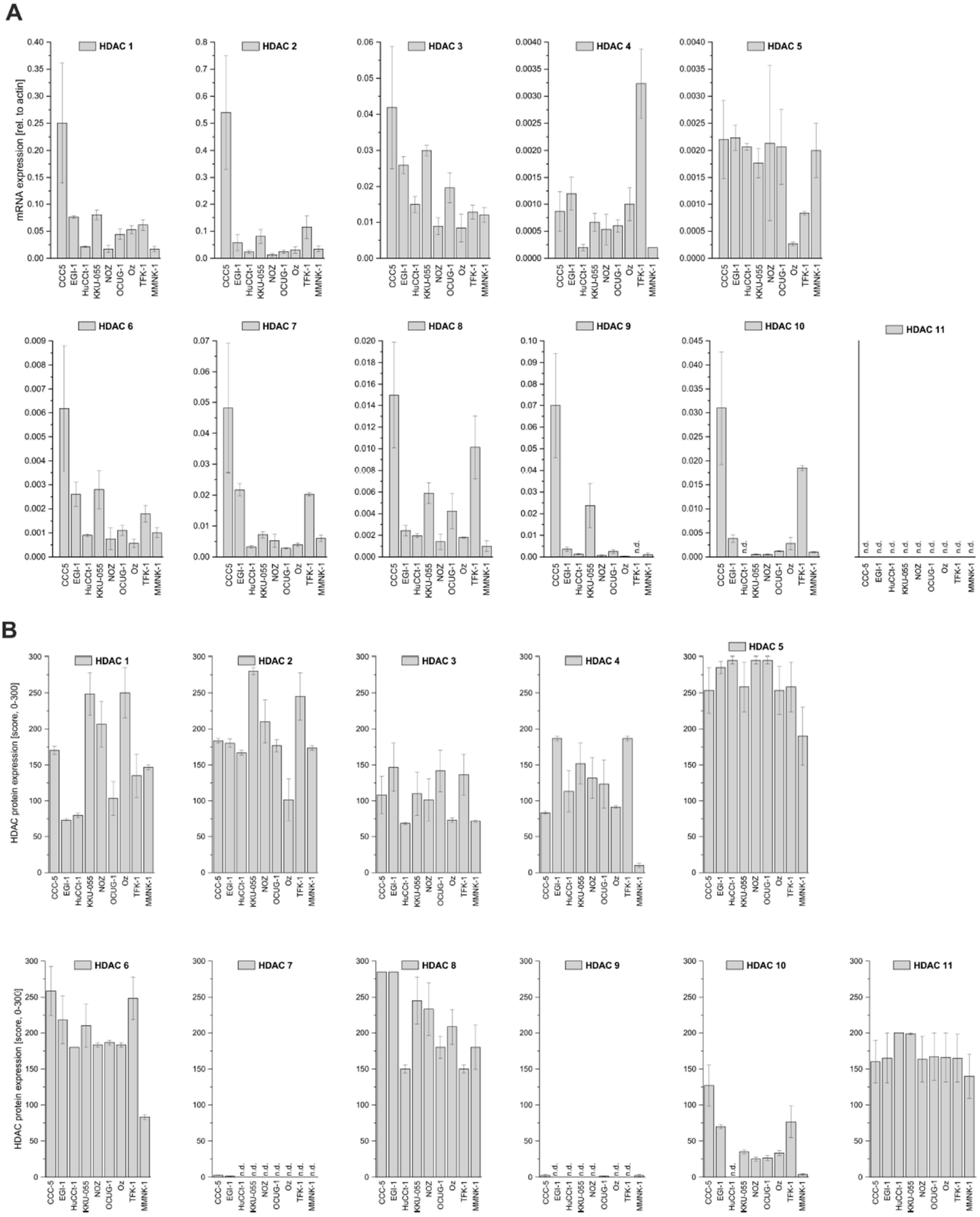

Figure S3. Expression profile of HDACs 1-11 in the used BTC in vitro model and non-tumor MMNK-1 cells. (**A**) mRNA levels. (**B**) Protein levels of HDACs 1-11 in BTC cell blocks and MMNK-1 cells measured by immunohistochemistry. Data are presented as mean value of at least three individual biological replicates. Abbreviations: BTC, biliary tract cancer; n.d., not detectable.

Belinostat [µM]	20.00	10.00	5.00	2.50	1.25	0.63	0.31	0.16	0.08	0.04
CCC-5	*	*			*					
EGI-1	*	*		*						*
HuCCT-1	*	*	*	*						**
KKU-055	**	**	**	**						
NOZ	**	**	**	*						
OCUG-1	**	**	**	**	**	**				
OZ	**	**	**	**	**					
TFK-1	**	**	**	**	*		*			*

LMK-235 [µM]	20.00	10.00	5.00	2.50	1.25	0.63	0.31	0.16	0.08	0.04
CCC-5										
EGI-1	*	*	*	*						
HuCCT-1	*	*	*					*		
KKU-055	**	**	**	**						
NOZ	**	**	**	*	*					
OCUG-1	*	**	**	*					**	
OZ	*	*	*							
TFK-1	*	*	*	**	**	**	*			

Mocetinostat [µM]	20.00	10.00	5.00	2.50	1.25	0.63	0.31	0.16	0.08	0.04
CCC-5	*	*								
EGI-1	*	*			**	**	**			
HuCCT-1	*							*		
KKU-055	**	**	**	**						
NOZ	**	**	**	**	*					
OCUG-1	**	**	**	**						
OZ										
TFK-1	**	**	**	**	*					*

Romidepsin [µM]	1.000	0.500	0.250	0.125	0.063	0.031	0.016	0.008	0.004	0.002
CCC-5										
EGI-1	*	*	*	*	*					
HuCCT-1	**	**	**	**	**	**	**	*		
KKU-055	**	**	**	**	**	**	**	**	*	
NOZ	**	**	**	**	**	**	**	**	**	**
OCUG-1	**	**	**	**	**	**	**	**	**	*
OZ	**	*	**	**	**	**	**	**	**	

**Romidepsin [nM]**	100.00	50.00	25.00	12.50	6.25	3.13	1.56	0.78	0.39	0.20
TFK-1	*	*	*	*	*	*				

**Tubastatin-A [µM]**	100.00	50.00	25.00	12.50	6.25	3.13	1.56	0.78	0.39	0.20
CCC-5	*		*	*						
EGI-1	**		*							
HuCCT-1	**	**	**	*						*
KKU-055	**	**	*							
NOZ	**	**								
OCUG-1	**	**								
OZ	**	**	**							
TFK-1	*									

**Vorinostat [µM]**	20.00	10.00	5.00	2.50	1.25	0.63	0.31	0.16	0.08	0.04
CCC-5										
EGI-1	*	*	*	*						
HuCCT-1	*						*			
KKU-055	**	**	*		*			*		
NOZ	**	**	*	*						
OCUG-1	**	**	**	*			**			
OZ	*									
TFK-1	**	*	**	*						

Figure S4. Statistics for Figures 2A-F. Significant differences in cell viability between treated and untreated samples were calculated by paired *t*-tests. * (light green) indicate significant ($p < 0.05$) and ** (dark green) indicate highly significant ($p < 0.01$) results, respectively.

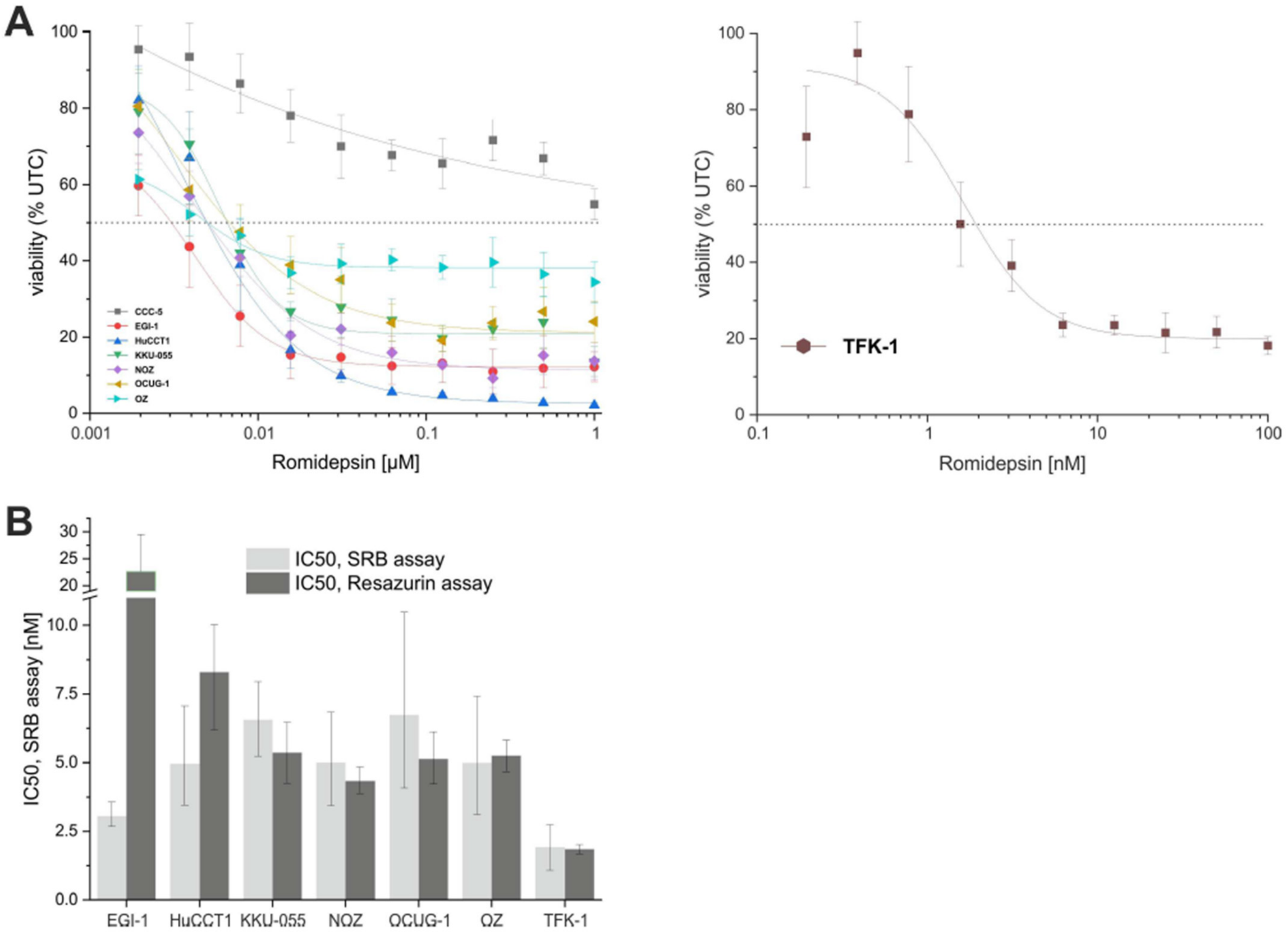

Figure S5. Reduction of cell viability of BTC cells by romidepsin using the SRB assay. Cells were seeded as described in the material and methods section. After 72 h of incubation with romidepsin, cells were fixed at 4 °C for 1 hour, washed and incubated with the SRB substrate for 15 min in the dark according to the manufacturer's protocol. After air-drying, samples were incubated with the solubilization solution and absorbance was measured at 565 nm using the Tecan Spark multimode reader. (**A**) BTC cells were incubated for 72 h with romidepsin. Shown are viability data related to untreated controls (UTC). (**B**) Comparison of IC_{50} values of the tested cell lines based on four parameter logistic regression. Data are presented as mean value ± SEM related to untreated control cells of at least three individual biological replicates. Abbreviations: BTC, biliary tract cancer.

Romidepsin [nM]		100.00	50.00	25.00	12.50	6.25	3.13	1.56	0.78	0.39	0.20
KKU-055	2 h										
	24 h	**	**	**	**	**	*	*			
	30 h	*	*	*	*	*	*	*			
	48 h	**	**	**	**	**					
TFK-1	2 h	*				*		*			
	24 h										
	30 h			*		*	*				
	40 h	**	**	**	**	**	**				

Figure S6. Statistics for Figures 4 A and B. Significant differences in cell viability between treated and untreated samples were calculated by paired *t*-tests. * (light green) indicate significant ($p < 0.05$) and ** (dark green) indicate highly significant ($p < 0.01$) results, respectively.

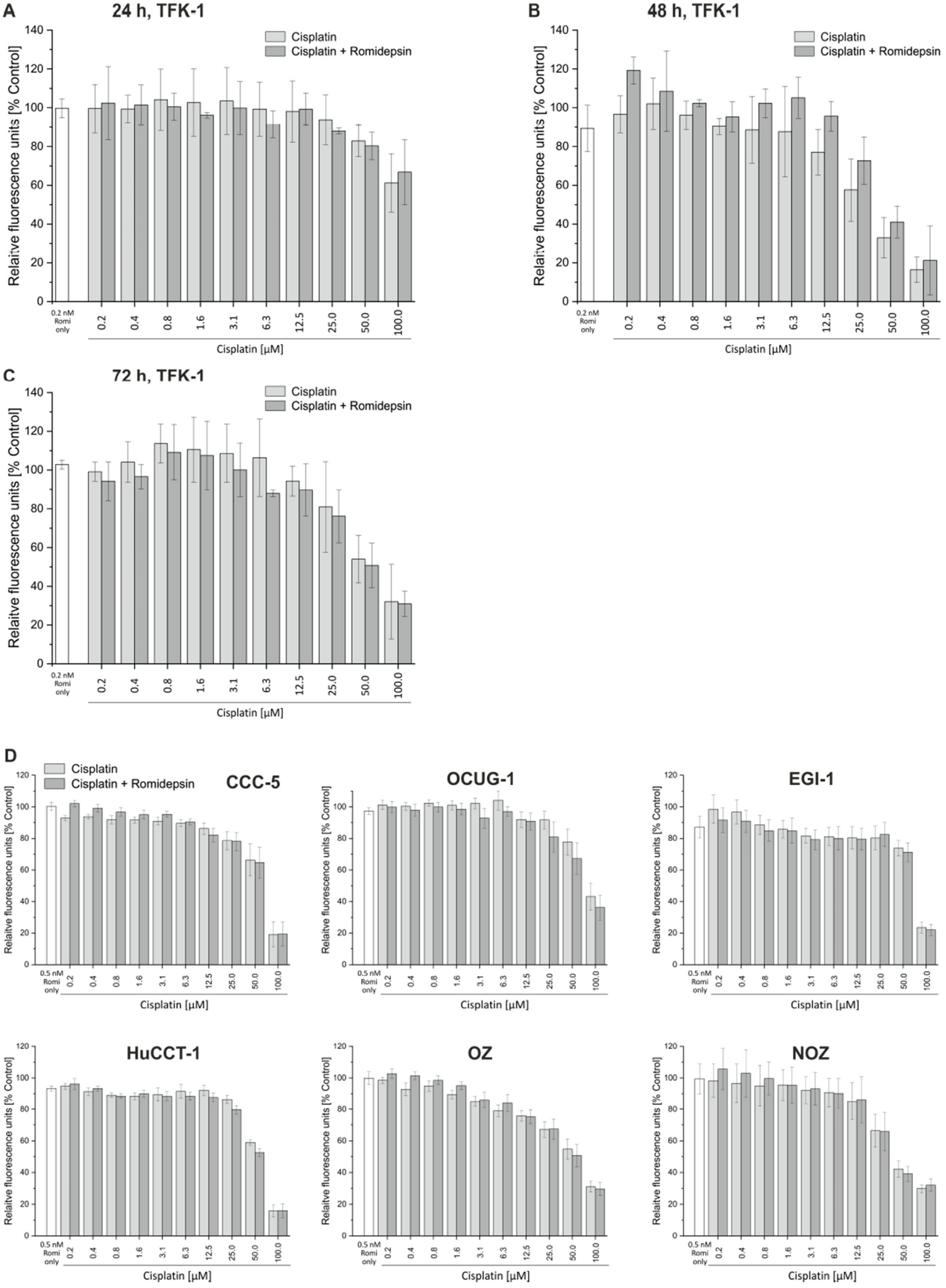
A
24 h, TFK-1
B
48 h, TFK-1
C
72 h, TFK-1
Cisplatin
Cisplatin + Romidepsin
Relaitve fluorescence units [% Control]
0.2 nM Romi only
Cisplatin [μM]
D
CCC-5
OCUG-1
EGI-1
HuCCT-1
OZ
NOZ
0.5 nM Romi only

Figure S7. Evaluation of combined romidepsin and cisplatin treatment. Simultaneous treatment of romidepsin-sensitive TFK-1 cells with a dilution series of cisplatin and 0.5 nM romidepsin for 24 h (**A**), 48 h (**B**) and 72 h (**C**), respectively. (**D**) Simultaneous treatment of BTC cells (without KKU-055 and TFK-1 cells) with romidepsin and cisplatin for 72 h. The left bar (white) represents the cytotoxic effect of 0.5 nM romidepsin as a single treatment. Data are presented as mean value of at least three individual biological replicates. Abbreviations: BTC, biliary tract cancer.

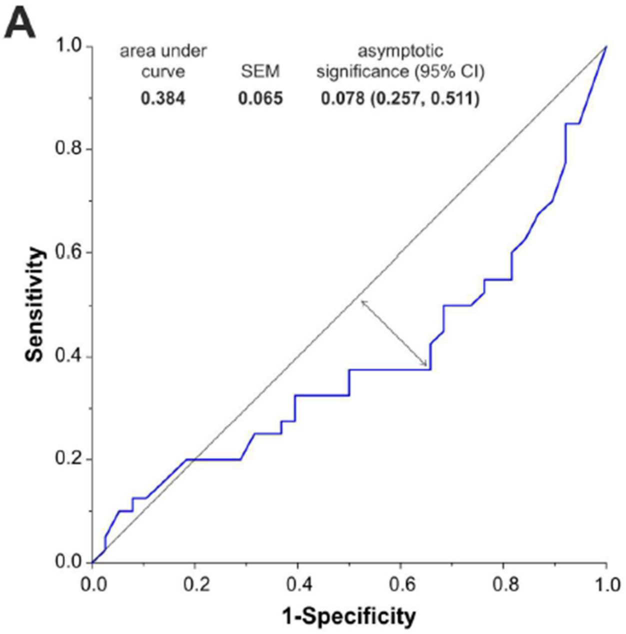

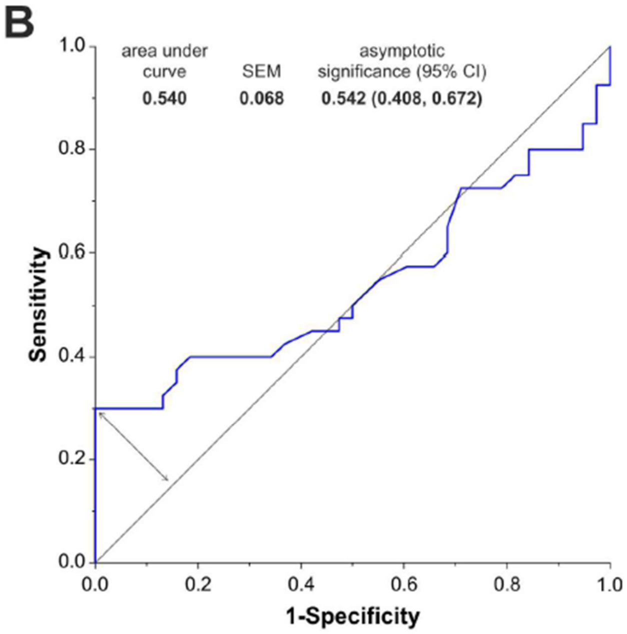

| J |	J	positive if ≥	sensitivity	1 - specificity
0.283	-0.283	70.000	0.375	0.658
0.266	-0.266	15.833	0.550	0.816
0.258	-0.258	63.333	0.400	0.658
0.257	-0.257	77.500	0.375	0.632
0.241	-0.241	14.167	0.575	0.816
0.239	-0.239	18.333	0.550	0.789
0.238	-0.238	27.500	0.525	0.763
0.237	-0.237	33.333	0.500	0.737
0.234	-0.234	43.333	0.450	0.684
0.233	-0.233	54.167	0.425	0.658
0.217	-0.217	10.000	0.625	0.842
0.216	-0.216	12.500	0.600	0.816
0.213	-0.213	22.500	0.550	0.763
0.204	-0.204	80.833	0.375	0.579
0.195	-0.195	5.833	0.700	0.895
0.193	-0.193	7.500	0.675	0.868
0.184	-0.184	38.333	0.500	0.684
0.178	-0.178	82.500	0.375	0.553
0.175	-0.175	123.333	0.325	0.500
0.151	-0.151	92.500	0.375	0.526
0.150	-0.150	113.333	0.350	0.500
0.146	-0.146	4.167	0.775	0.921
0.125	-0.125	104.167	0.375	0.500
0.120	-0.120	151.667	0.275	0.395
0.118	-0.118	157.500	0.250	0.368
0.097	-0.097	0.833	0.850	0.947
0.096	-0.096	2.667	0.825	0.921
0.096	-0.096	133.333	0.325	0.421
0.095	-0.095	146.667	0.300	0.395
0.093	-0.093	154.167	0.275	0.368
0.092	-0.092	163.333	0.250	0.342
0.089	-0.089	173.333	0.200	0.289
0.071	-0.071	1.833	0.850	0.921
0.070	-0.070	141.667	0.325	0.395
0.066	-0.066	168.333	0.250	0.316
0.047	0.047	213.333	0.100	0.053
0.046	0.046	204.167	0.125	0.079
0.037	-0.037	180.000	0.200	0.237
0.024	0.024	225.833	0.050	0.026
0.021	0.021	210.000	0.100	0.079
0.020	0.020	195.000	0.125	0.105
0.018	0.018	188.333	0.150	0.132
0.016	0.016	185.000	0.200	0.184
0.001	-0.001	243.333	0.025	0.026
0.000	0.000	-1.000	1.000	1.000
0.000	0.000	251.000	0.000	0.000

| J |	J	positive if ≥	sensitivity	1 - specificity
0.300	0.300	262.500	0.300	0.000
0.274	0.274	257.500	0.300	0.026
0.250	0.250	267.500	0.250	0.000
0.247	0.247	252.500	0.300	0.053
0.225	0.225	272.500	0.225	0.000
0.221	0.221	247.500	0.300	0.079
0.217	0.217	207.500	0.375	0.158
0.216	0.216	195.000	0.400	0.184
0.193	0.193	224.167	0.325	0.132
0.192	0.192	216.667	0.350	0.158
0.189	0.189	187.500	0.400	0.211
0.168	0.168	237.500	0.300	0.132
0.163	0.163	182.500	0.400	0.237
0.147	-0.147	14.167	0.800	0.947
0.137	0.137	176.667	0.400	0.263
0.125	0.125	277.500	0.125	0.000
0.124	-0.124	7.500	0.850	0.974
0.122	-0.122	12.500	0.825	0.947
0.097	-0.097	10.000	0.850	0.947
0.095	-0.095	17.500	0.800	0.895
0.092	-0.092	34.167	0.750	0.842
0.084	-0.084	80.833	0.600	0.684
0.084	0.084	171.667	0.400	0.316
0.083	-0.083	88.333	0.575	0.658
0.075	-0.075	3.667	0.925	1.000
0.074	-0.074	6.000	0.900	0.974
0.068	-0.068	21.667	0.800	0.868
0.067	-0.067	30.833	0.775	0.842
0.066	-0.066	40.000	0.750	0.816
0.064	-0.064	45.833	0.725	0.789
0.059	-0.059	74.167	0.625	0.684
0.058	0.058	165.000	0.400	0.342
0.057	-0.057	91.667	0.575	0.632
0.057	0.057	158.333	0.425	0.368
0.050	-0.050	2.667	0.950	1.000
0.050	0.050	282.500	0.050	0.000
0.049	-0.049	4.667	0.925	0.974
0.042	-0.042	25.833	0.800	0.842
0.038	-0.038	48.333	0.725	0.763
0.034	-0.034	70.000	0.650	0.684
0.030	-0.030	96.667	0.575	0.605
0.029	0.029	155.000	0.450	0.421
0.025	-0.025	1.000	0.975	1.000
0.025	-0.025	133.333	0.475	0.500
0.024	-0.024	145.000	0.450	0.474
0.014	0.014	61.667	0.725	0.711
0.012	-0.012	53.333	0.725	0.737
0.003	0.003	151.667	0.450	0.447
0.003	-0.003	105.000	0.550	0.553
0.001	-0.001	118.333	0.525	0.526
0.001	0.001	138.333	0.475	0.474
0.000	0.000	-1.000	1.000	1.000
0.000	0.000	128.333	0.500	0.500
0.000	0.000	286.000	0.000	0.000

Figure S8. ROC analysis. For HDAC 1 (**A**) and HDAC 2 (**B**), the receiver operating characteristics (ROC) were used for determination of a (discrimination) HDAC expression threshold separating clinical cases according to their survival status including the Youden Index (J) indicating the threshold with the highest discrimination power.

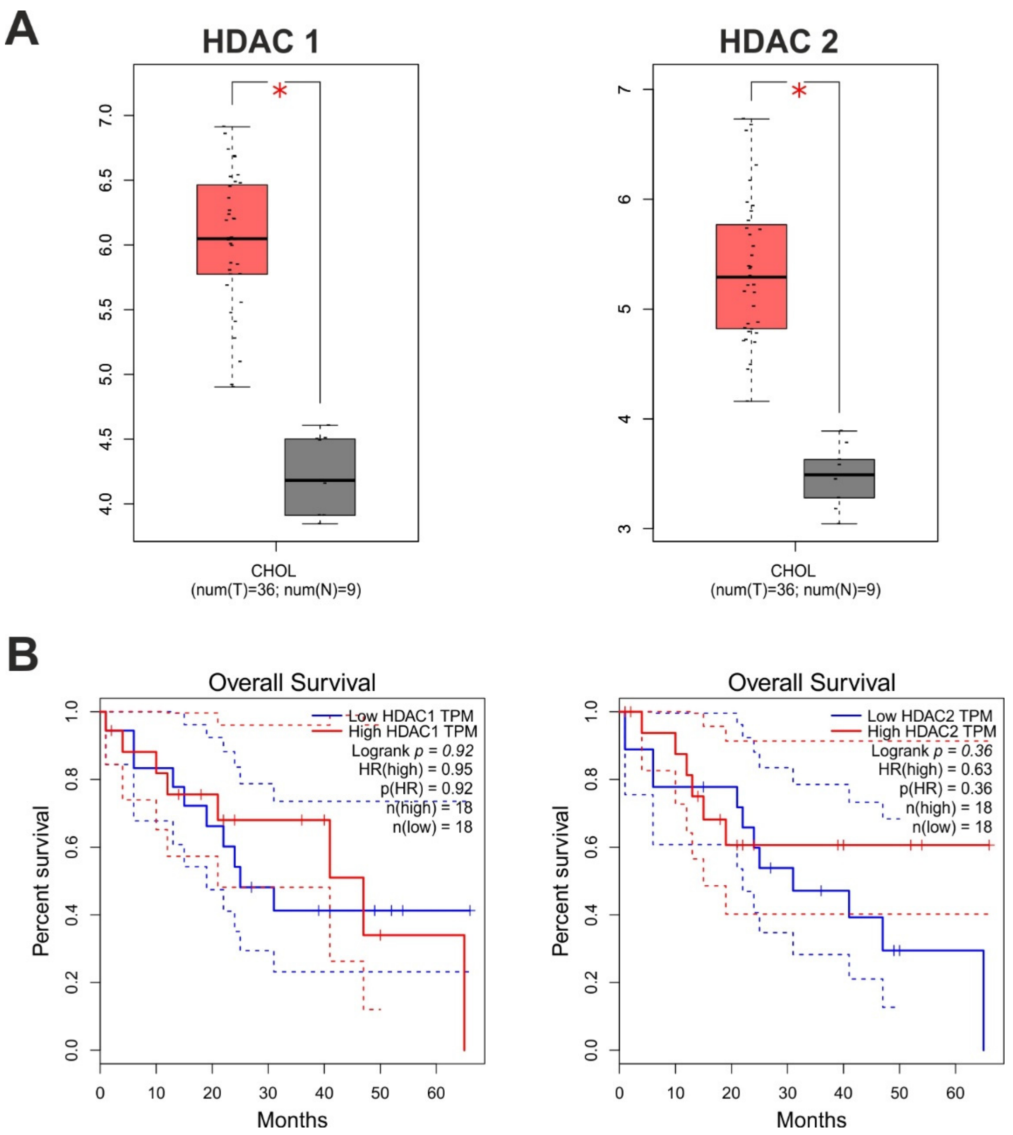

Figure S9. In silico analysis for HDAC 1 and HDAC 2 expression / survival data using GEPIA ((http://gepia.cancer-pku.cn accessed date 06/2021; see Tang, Z. et al. (2017) GEPIA: a web server for cancer and normal gene expression profiling and interactive analyses. Nucleic Acids Res, 10.1093/nar/gkx247). (**A**) HDAC 1 and HDAC 2 are overexpressed in BTC tissue compared to adjacent non-tumor tissue. (**B**) Survival analysis (default cut-off value = median HDAC 1/2 score).

(**A**)

Step	HDAC1-Cutoff	Logistic regression, p-value	"positive" cases (*n*)
0	0	0.226	34
1	5	0.016	28
2	10	0.014	25
3	15	0.012	22
4	20	0.027	22
5	25	0.019	21
6	30	0.013	20
7	35	0.013	20
8	40	0.025	18
9	45	0.025	18
10	50	0.038	17
11	55	0.038	17
12	60	0.038	17
13	65	0.019	15
14	70	0.019	15
15	75	0.034	15
16	80	0.078	15
17	85	0.117	15
18	90	0.117	15
19	95	0.117	15
20	100	0.117	15
21	105	0.169	15
22	110	0.117	14
23	115	0.117	14
24	120	0.12	13
25	125	0.12	13
26	130	0.304	13
27	135	0.304	13
28	140	0.444	13
29	145	0.395	12
30	150	0.329	11
31	155	0.48	10
32	160	0.518	10
33	165	0.518	10
34	170	0.348	8
35	175	0.348	8
36	180	0.692	8
37	185	0.988	8
38	190	0.696	5
39	195	0.696	5
40	200	0.492	5
41	205	0.492	5
42	210	0.658	4
43	215	0.67	2
44	220	0.67	2
45	225	0.67	2
46	230	0.67	2
47	235	0.67	2
48	240	0.738	1
49	245	0.738	1
50	250		0
51	255		0
52	260		0
53	265		0
54	270		0
55	275		0
56	280		0
57	285		0
58	290		0
59	295		0
60	300		0

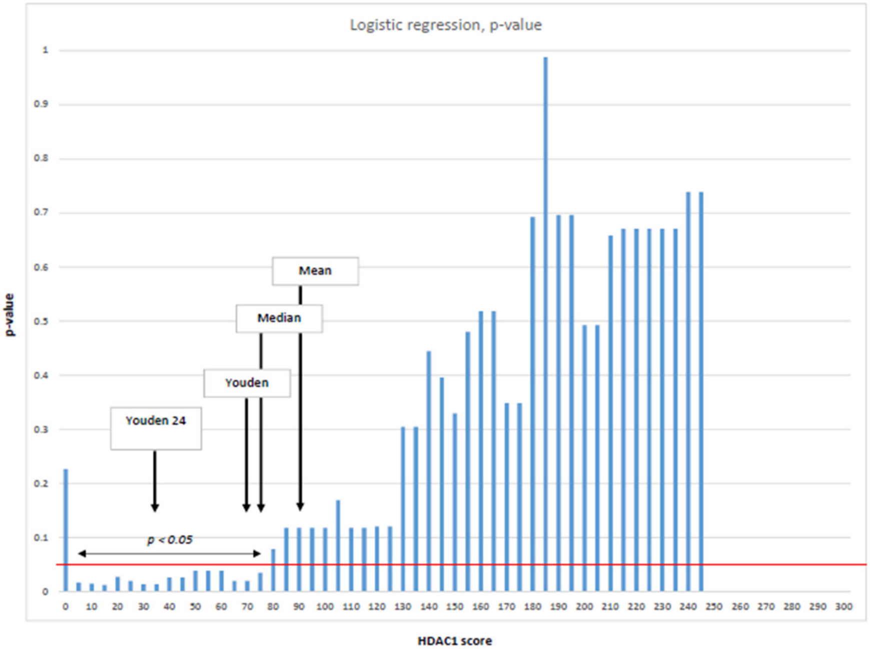

(**B**)

Step	HDAC2-Cutoff	Logistic regression, p-value	"positive" cases (*n*)
0	0	0.31	39
1	5	0.453	37
2	10	0.452	34
3	15	0.702	32
4	20	0.827	32
5	25	0.951	32
6	30	0.684	31
7	35	0.79	30
8	40	0.79	30
9	45	0.72	29
10	50	0.991	29
11	55	0.991	29
12	60	0.824	29
13	65	0.824	29
14	70	0.781	26
15	75	0.418	24
16	80	0.418	24
17	85	0.418	24
18	90	0.409	23
19	95	0.588	23
20	100	0.768	22
21	105	0.768	22
22	110	0.717	21
23	115	0.717	21
24	120	0.717	21
25	125	0.717	21
26	130	0.49	19
27	135	0.49	19
28	140	0.582	18
29	145	0.582	18
30	150	0.696	18
31	155	0.956	18
32	160	0.507	16
33	165	0.507	16
34	170	0.34	16
35	175	0.194	16
36	180	0.173	16
37	185	0.129	16
38	190	0.073	16
39	195	0.073	16
40	200	0.097	15
41	205	0.097	15
42	210	0.097	15
43	215	0.16	14
44	220	0.11	13
45	225	0.11	13
46	230	0.154	12
47	235	0.154	12
48	240	0.154	12
49	245	0.003	12
50	250	0.002	12
51	255	0	12
52	260	0	12
53	265	0	10
54	270	0.001	9
55	275	0.03	5
56	280	0.309	2
57	285		0
58	290		0
59	295		0
60	300		0

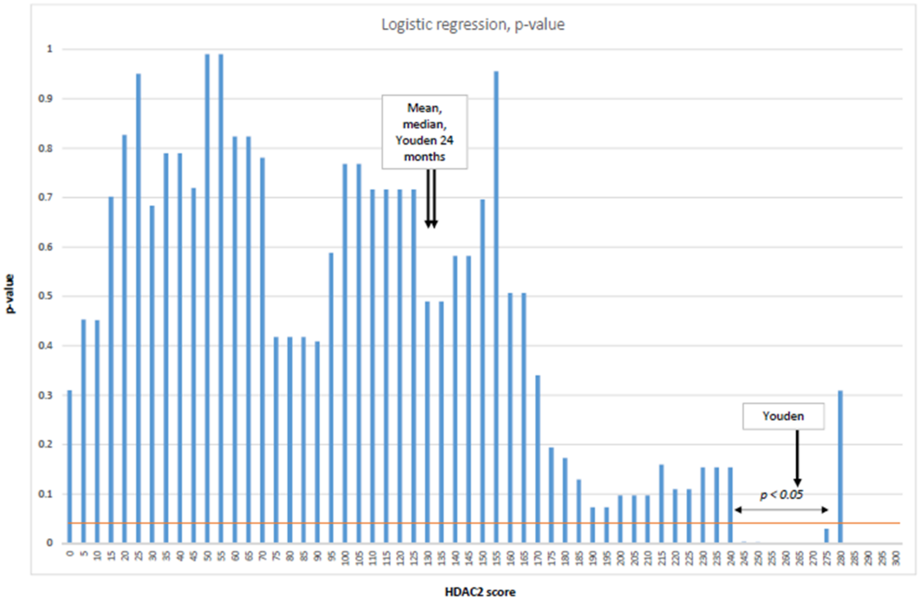

Figure S10. Calculation of logistic regression-derived p-values for HDAC 1 (**A**) and HDAC 2 (**B**). The graphs indicate the high discrimination power of the calculated Youden-index as well as the discrimination potential of the time-dependent mean and median values (t = 24 months) for HDAC 1 (**A**) and HDAC 2 (**B**), respectively.

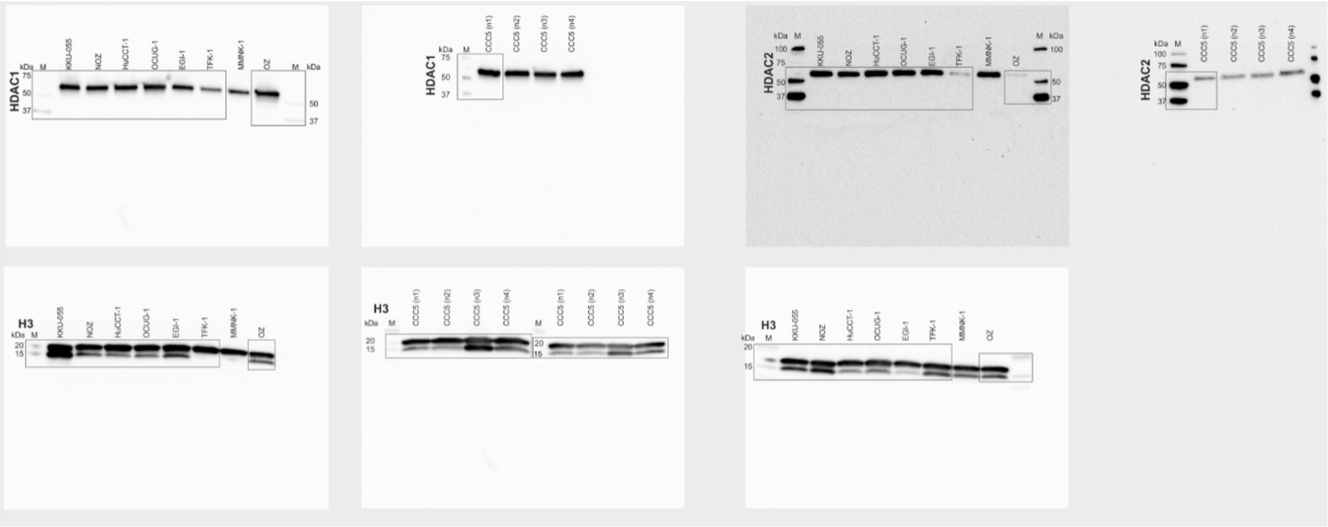

Figure S11. Original (uncropped) blots for Figure 3.

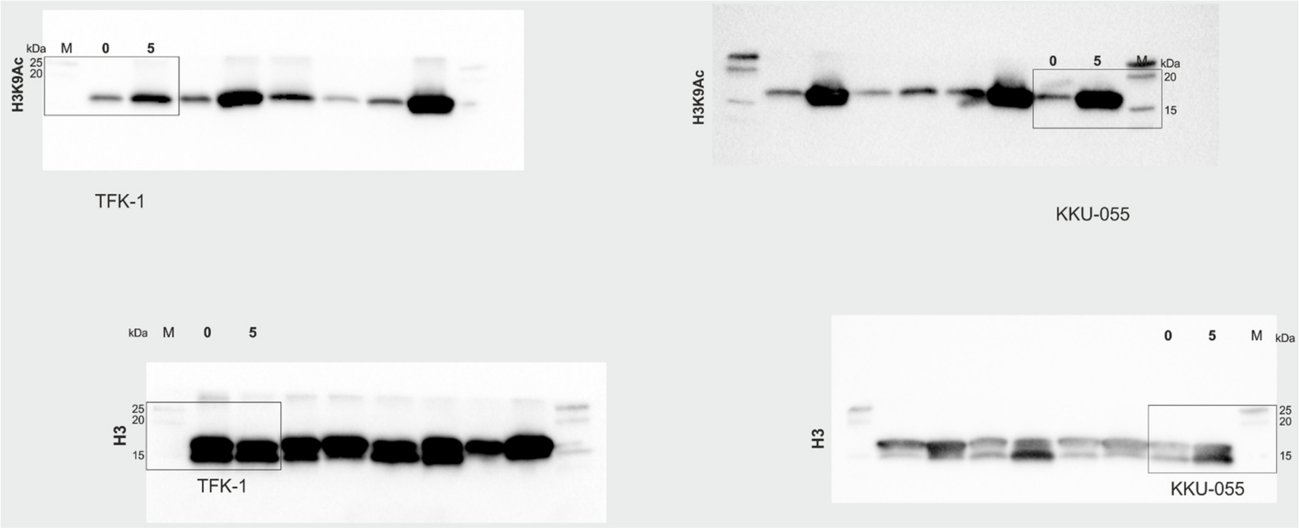

Figure S12. Original (uncropped) blots for Figure 5.
